# Ionizing and nonionizing radiations can change physicochemical, technofunctional, and nutritional attributes of starch

**DOI:** 10.1016/j.fochx.2023.100771

**Published:** 2023-06-24

**Authors:** Hadis Rostamabadi, Ilkem Demirkesen, Bengi Hakgüder Taze, Asli Can Karaca, Mehvish Habib, Kulsum Jan, Khalid Bashir, Monica R. Nemțanu, Rosana Colussi, Seid Reza Falsafi

**Affiliations:** a Nutrition and Food Security Research Center, Isfahan University of Medical Sciences, Isfahan 81746–73461, Iran; b Department of Animal Health, Food and Feed Research, General Directorate of Agricultural Research and Policies, Ministry of Agriculture and Forestry, Ankara, Turkey; c Usak University, Faculty of Engineering, Department of Food Engineering 1 Eylul Campus, 64000 Usak, Turkey; d Department of Food Engineering, Faculty of Chemical and Metallurgical Engineering, Istanbul Technical University, 34469 Istanbul, Turkey; e Department of Food Technology, Jamia Hamdard, New Delhi 110062, India; f Electron Accelerators Laboratory, National Institute for Laser, Plasma and Radiation Physics, 409 Atomiștilor St., P.O. Box MG-36, 077125 Bucharest-Măgurele, Romania; g Center for Chemical, Pharmaceutical and Food Sciences, Federal University of Pelotas, Pelotas, Campus Universitário, s/n, 96010-900, Pelotas, RS, Brazil; h Isfahan Endocrine and Metabolism Research Center, Isfahan University of Medical Sciences, Isfahan, Iran

**Keywords:** Starch, Modification, Ionizing radiation, Non-ionizing radiation, Physicochemical attributes, Nutritional behavior

## Abstract

Challenges for the food/non-food applications of starch mostly arise from its low stability against severe processing conditions (i.e. elevated temperatures, pH variations, intense shear forces), inordinate retrogradability, as well as restricted applicability. These drawbacks have been addressed through the modification of starch. The escalating awareness of individuals toward the presumptive side effects of chemical modification approaches has engrossed the attention of scientists to the development of physical modification procedures. In this regard, starch treatment via ionizing (i.e. gamma, electron beam, and X-rays) and non-ionizing (microwave, radiofrequency, infrared, ultraviolet) radiations has been introduced as a potent physical strategy offering new outstanding attributes to the modified product. Ionizing radiations, through dose-dependent pathways, are able to provoke depolymerization or cross-linking/grafting reactions to the starch medium. While non-ionizing radiations could modify the starch attributes by changing the morphology/architecture of granules and inducing reorientation/rearrangement in the molecular order of starch amorphous/crystalline fractions.

## Highlights

• Gamma irradiation and microwave were the most investigated methods.
• Irradiations mostly impart physical changes into starch granules.
• Gamma irradiation at high doses could provoke chemical reactions in molecules.
• Uncontrolled doses irradiation could adversely affect the starch-based products.

## Graphical abstract

**Figure:**
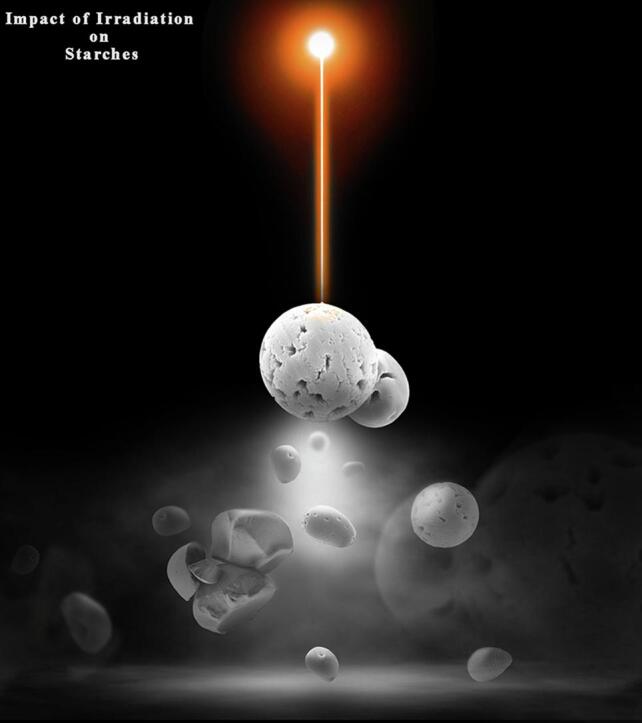

## 1 Introduction

**Fig. 1:**
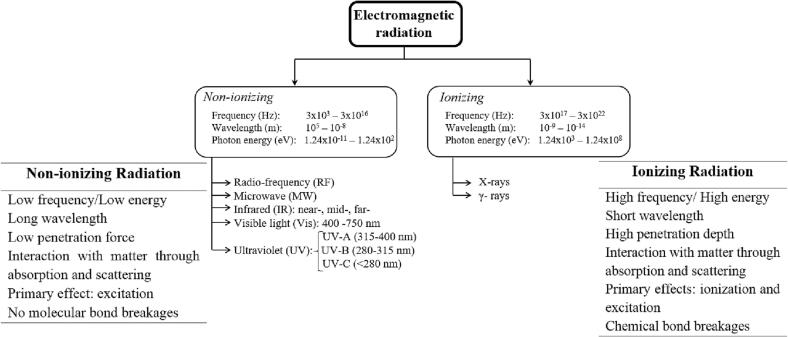
Electromagnetic waves.

Starch is the second major biopolymer of the world's landmass. Apart from its naturally occurring presence in foods, starch has been involved in various food/pharma formulations to endow novel promising characteristics to the fabricated products. However, the widespread application of starch has been restricted by some limitations including its low shear/thermal resistibility, as well as retrogradability. Presently, starch modification through chemical (e.g. cross-linking, etherification, esterification, and oxidation) physical (i.e. thermal and non-thermal) and enzymatic techniques has been the most developed approach to come across such shortcomings (Aaliya et al., 2021). Apart from being fast and efficacious in altering the starch attributes, the implementation of chemical modifications is occasionally accompanied by some adverse issues regarding the presence of detrimental chemical residues in the final product, upsurging the consumer's solicitation for safer alternatives (Rostamabadi et al., 2022). Physical modifications are the safe surrogates for chemical modification approaches leading to the generation of novel starches, chemically/architecturally different from the native counterparts, with improved viscoelasticity, water/oil holding capacity, freeze–thaw stability, as well as less retrogradability without leaving chemical residues within the modified product (Barroso et al., 2019; Bashir and Aggarwal, 2019). Within the range of physical modification methods, radiation processings (including ionizing and non-ionizing irradiations) have attained a seat at the table by virtue of their promising features e.g. cost-effectiveness, non-toxicity, tuneability, as well as scalability. More so, these eco-friendly techniques are typically performed at low/moderate temperatures rendering the minimum changes in the structure of thermosensitive bioactive ingredients (Kanatt, 2020). In this category, ionizing radiations (accelerated electron beams, gamma, and X-rays) could cause the generation of various free radical species within the starch medium (Fig. 1). These radicals could further interact with starch molecules leading to radiolysis, and cross-linking/grafting reactions each provoking a diverse range of alterations in the structural/physicochemical characteristics of treated starch (Kong et al., 2016, Lam et al., 2021). On the contrary, non-ionizing radiations (e.g. microwave, radio frequency, ultraviolet, and infrared emissions) could efficaciously modify starch characteristics without manipulating the glycosidic backbone of the starch molecules. Nevertheless, it should be noted that the extent of modifications upon both ionizing and non-ionizing strategies are mainly governed by the type of radiation, radiation condition (i.e. dose, dose rate, time, and temperatures), as well as the type/characteristics of starch (Ma et al., 2020).

Recent years have witnessed the increasing focus of researchers on developing promising modified starches through their exposure to ionizing/non-ionizing radiations. More interestingly the emergence of new starches extracted from new sources has drawn the attention of scientists to explore their behavior under various irradiation conditions. Thereby, the main focus of this review was the recent advances in the impacts of ionizing/non-ionizing radiations on various features (i.e. chemical composition, morphology, and structure) of starches extracted from conventional and novel sources.

## 2 Different types of radiation

### 2.1 Ionizing radiation

The most applied ionizing radiation types to process/modify biomacromolecules are gamma rays (electromagnetic radiation) and electron beam (high-energy particle radiation). Gamma rays are emitted by radionuclides such as isotopes of cobalt-60 (^60^Co) and cesium-137 (^137^Cs). On the contrary, electron beam consists of accelerated electrons, which are charged particles produced by regular electricity using usually linear accelerators that do not involve sources of radioactive isotopes (Brașoveanu & Nemțanu, 2017). It should be noted herein that gamma processing and electron beam irradiation interact essentially in a similar way with materials that are exposed to irradiation. The major differences of these two radiation types in terms of applicability are actually related to the penetration power and dose rate. Gamma radiation possesses higher penetration capability and can be used for thick//dens materials while electron beam has limited penetration depth with significantly higher dose rates. The elementary processes involved in the γ-ray energy transfer include mainly the photoelectric effect, Compton scattering, the generation of electron–positron pairs, which cause the release of fast electrons that lose energy through the same effects as the accelerated electrons of the electron beam. Then, two distinct primary effects, ionization and excitation of atoms and molecules of the matter take place. In this way, ions, free radicals (highly reactive entities) and excited molecules are formed. Further, these primary species are involved in different reactions such as dissociation of excited molecules, radical–radical or radical-molecule recombination, leading to secondary effects (Rostamabadi et al., 2022).

The most important radiation quantities are the absorbed dose and the absorbed dose rate (IAEA, 1987). The absorbed dose, *D*, expressed in Gy, is the amount of energy absorbed per unit mass of irradiated matter at a point in the region of interest and the absorbed dose rate, $\dot{D}$, expressed in Gy/s, is defined as the rate of change of the absorbed dose with time (Brașoveanu & Nemțanu, 2017).

### 2.2 Non-ionizing radiation

Among the non-ionizing radiation types, the radio-frequency, microwaves and ultraviolet light are frequently utilized in the processing/modification of biomacromolecules. These types of radiations are usually produced by a machine or an instrument (Bisht et al., 2021), which cause the vibration of atoms in the matter, generating generally the heating as a main effect. For instance, the principal effect of microwave interaction with a biomaterial is the dielectric heating (Braşoveanu and Nemţanu, 2014, Oyeyinka et al., 2021). The energy is absorbed by target material that contain polar groups and generates heat by orienting the polarization (Schnabel, 2014). To be more precise, only polar materials that contains molecules with a permanent dipole moment as well as materials having mobile ions are able to absorb such energy, while non-polar materials can be considered transparent and are not affected (Schnabel, 2014). It is also noteworthy herein that microwave propagation in matter depends strongly on the dielectric and the magnetic properties of the material. These properties are essential factors in microwave processing/modification of biomacromolecules, being highly dependent on the type of biomacromolecules, moisture content, temperature and microwave frequency (Braşoveanu & Nemţanu, 2014). Another effects that are highly visible especially in the case of ultraviolet light is the excitation of chromophores present in the macromolecule, if any, and a change in the charge distribution in the molecule as well (Kowalonek, 2017, Schnabel, 2014). It should be noted that practically ultraviolet light is a bridge between non-ionizing and ionizing radiation as it lies next to ionizing radiation in the electromagnetic spectrum (Rostamabadi et al., 2022c).

Radiation has many advantages in material processing, being a useful mean to induce changes in structure and functional properties of materials without environmental negative implication. Irradiation is an ecological process that involves no use of pollutants, no production of waste, no penetration of toxic substances into treated products/materials. Therefore, the effects of irradiation on biological molecules are a result of direct action and/or indirect action of radiation. The major factors contributing to the radiation-induced effects on biological systems could be classified as i) Macromolecule Factors- type of macromolecule, water content, density, dielectric properties, penetration depth, temperature, and ii) Radiation Processing parameters - radiation type, radiation source, process variables (energy, power, current), system geometry, oxygen presence, exposure time.

## 3 Impacts of ionizing radiations on starch

Numerous researches have explored the use of ionizing radiations to prevent food microbial deterioration without compromising the food safety, quality, or nutritional content (Bisht et al., 2021; Fallah et al., 2022; Munir and Federighi, 2020; Fernandes et al., 2017). However, the impact of such non-thermal treatments on different component of foods (i.e. starch) is inevitable. In this section the impact of ionizing radiations on various features of starches has been discussed.

### 3.1 Impact of ionizing radiation on chemical composition and molecular structure of starch

Reportedly, the foods exposed to radiation up to 60 kGy are both nutritionally suitable and safe (Munir and Federighi, 2020). However, the molecular structure and composition of their constituents, particularly those of starch, proteins, vitamins, and lipids, could be altered upon irradiation. For instance, Kumar et al., (2017) reported a reduction in apparent amylose content of brown rice starch treated with different doses of gamma rays (5, 10, 15, and 20 kGy). They ascribed this result to the breakage of amylose chains upon irradiation. Gamma irradiation also resulted in a decrease in pH of starch solutions due to the conversion of starch molecules into short chain carboxylic acids. In another attempt Gul et al., (2016) studied the impact of 0, 2, 5 and 10 kGy gamma rays on rice starch. They obtained a reduction in moisture content of irradiated starches due to the ionization/evaporation of water molecules when affected by γ-rays. A remarkable reduction in apparent amylose content, along with an increase in pH and carboxyl content indicated the breakage of amylose fractions into residues with carboxylic functional groups. These alteration in apparent amylose content, pH and carboxyl residues were also reported for γ-irradiated talipot starch (Aaliya et al., 2021), chickpea starch (Bashir et al., 2017), and lotus seed starch (Punia et al., 2020). However, when compared with other compartment of starch granule, amylopectin is the most affected constituent upon irradiation. According to high performance size exclusion chromatography (HPSEC) experiments, irradiation could result in: i) the depolymerization of amylopectin into fractions of lower molecular weight through its reaction with free-radical species upon molecular radiolysis phenomenon (Atrous et al., 2015, Castanha et al., 2019, Polesi et al., 2016). ii) an increase in content of chains with DP of 6–12, and iii) a decrease in the extent of branches with DPs > 36 (Chung and Liu, 2009, Polesi et al., 2016). Nevertheless, these researchers have corroborated that lower doses of gamma irradiation (<10 kGy) barely affect the structure of amylopectin. Furthermore, gamma irradiation could provoke the formation of small amylopectin like structures upon cross-linking reactions (Bao et al., 2005). The extent of these changes are mostly governed by the type of starch and the intensity of gamma rays. (Polesi et al., 2016) reported that lower dose of gamma irradiation, 0.4 kGy, is more capable of inducing cross-linking reactions while greater doses are favorable for radiolysis and depolymerization reactions.

**Fig. 2:**
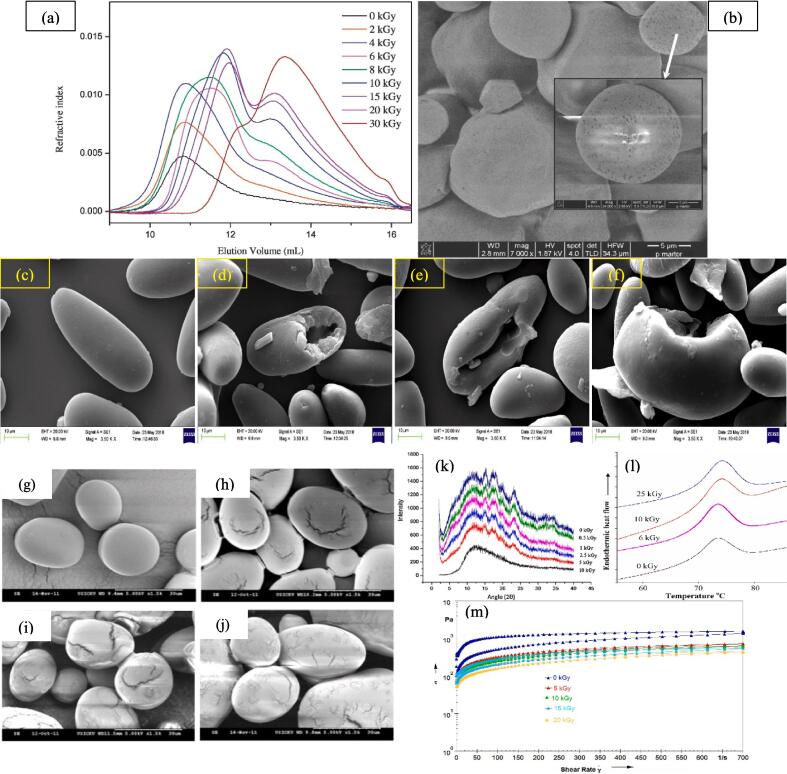
Elution behavior of the gamma irradiated starch and normal waxy maize starch assessed by HPSEC-MALLIS-RI (a) reprinted with permission from (Zhou et al., 2020), SEM micrographs of corn starch granule treated with 50 kGy Gamma rays (b) reprinted with permission from (Braşoveanu et al., 2013), SEM micrographs of native and gamma irradiated kithul starch at 0 kGy (c), 2.5 kGy (d), 5 kGy (e) and 10 kGy (f), reprinted with permission from (Sudheesh et al., 2019), SEM micrographs of gamma irradiated red potato starches: (g – 0 kGy), (h – 5 kGy), (i – 10 kGy), (j – 20 kGy), the white bar is equal to 30 µ, magnification: ×1500, reprinted with permission from (Gani et al., 2014), XRD patterns of native and gamma irradiated chickpea starches (k), reprinted with permission from (Bashir & Aggarwal, 2017), DSC thermograms of native (0 kGy) and gamma irradiated (6, 10, 25 kGy) sago starch(l), reprinted with permission from (Othman et al., 2015), and Shear stress-shear rate curves of native and irradiated oat starch, reprinted with permission from. (For interpretation of the references to color in this figure legend, the reader is referred to the web version of this article.)

Numerous attempts have also investigated the impact of electron beam (EB) radiation on starch molecules. In a recent attempt (Zhou et al., 2020) studied the molecular structure of electron beam treated waxy maize starch (2–30 kGy) and obtained a decrease in molecular weight and branch chain length of starch with elevating the radiation dose (Fig. 2**a**). They suggested that α-1,6-glycosidic linkages are more prone to breakage than α-1,4-glycosidic bonds resulting in the formation of short chain amylose-like structures. In another study the molecular structure of EB treated rice starch was assessed by (Pan et al., 2020). Their HPSEC results revealed that low dose of EB (<4 kGy) were not potent enough to induce change in molecular structure of starch, however, long starch chains could be notably degraded to short linear chains upon high energy EB treatments (4–10 kGy). They also suggested that EB irradiation mostly change the amorphous regions while starch molecules located within crystalline fractions could tolerate the radiation even at elevated energy levels. (Braşoveanu et al., 2013) outlined that EB treatment had no remarkable impact on the moisture, ash and protein content of corn starch. However, this treatment reduced the starch fat content and promoted the generation of carboxylic residues mainly through free-radical induced radiolysis phenomenon. An overview of the change in molecular structure and chemical composition of starch upon treatment with ionizing radiation is provided in Table 1.

**Table 1:** **-** an overview on the effect of ionizing radiations on physicochemical and techno-functional attributes of various starches.

**Type of radiation**	**Starch**	**Radiation condition**	**Thermal properties**	**XRD**	**Swelling and Solubility**	**Digestibility**	**Pasting and rheology**	**Morphology of granules**	**Reference**
Gamma rays	brown rice starch	5–20 kGy	To, Tp, Tc ↓ ΔH ↓	A type RC ↓ with dosage	SP ↓ SI ↑	–	paste attributes ↓	Not changed	Kumar et al., (2017)
	chickpea starch	0, 0.5, 1, 2.5, 5 and 10 kGy	To, Tp, Tc ↓ ΔH ↓	C type RC ↓ with dosage	SP ↑ SI ↑	–	paste attributes ↓	slight surface fissures created	Bashir & Aggarwal, (2017)
	rice starch	0, 2, and 10 kGy	Not changed ΔH ↓ at 10 kGy	A type RC ↓ with dosage	SP ↓ SI ↑	–	paste attributes ↓	Not changed	Gul et al., (2016)
	rice starch	0, 5, 10, and 20 kGy	–	A type RC ↓ with dosage	SP ↓ SI ↑	–	paste attributes ↓	crack generation and deformation	Ashwar et al., (2014)
	maize starch	1, 2, 5, 10, 20, 50, 100, 200, 500 kGy	To, Tp, Tc ↓ ΔH ↓	A type Slight decrease in RC	SP ↓ SI ↑	–	paste attributes ↓	Not changed	Liu et al., (2012)
	bean starch	5, 10 and 20 kGy	–	C type RC ↓ with dosage	SP ↓ SI ↑	–	paste attributes ↓	Granule destruction at highest dose	Gani et al., (2012)
	Indian Horse Chestnut	0, 5, 10, 15 kGy	–	A type RC ↓ with dosage	SP ↓ SI ↑	–	paste attributes ↓	Not changed	I. A. Wani et al., (2014)
	sago starch	6, 10 and 25 kGy	To and Tp ↑ Tc and ΔH not changed	A type RC ↓ with dosage	SP ↓ SI ↑	–	–	Not changed	Othman et al., (2015)
	broad bean starch	0, 5, 10 and 15 kGy	–	C type RC ↓ with dosage	SP ↓ SI ↑	–	paste attributes ↓	fissures appeared	Sofi et al., (2013)
	kithul starch	0.5, 1, 2.5, 5, 10 kGy	To, Tp, Tc ↑ ΔH ↑	A type RC ↓ with dosage	SP ↓ SI ↑	RS ↓ Digestibility ↑	paste viscosities ↓	fissures & cracks appeared	Sudheesh et al., (2019)
	buckwheat	5, 10, 15 and 20 kGy	To, Tp, Tc ↓ ΔH ↓	A type RC ↓ with dosage	SP ↓ SI ↑		paste attributes ↓	Not changed	Dar et al., (2018)
	oat	5, 10, 15 and 20 kGy	To, Tp, Tc ↓ ΔH ↓	A type RC ↓ with dosage	SP ↓ SI ↑		paste attributes ↓	Not changed	Dar et al., (2018)
	wheat	0.5, 1, 2.5, 5 and 10 kGy	–	–	SP ↑ SI ↑	–	paste viscosities ↓	–	Bashir et al., (2017)
	*Amorphophallus* starch	5, 10, 15, 20 and 25 kGy	To, Tp, Tc ↑ ΔH ↑	C type RC ↓ with dosage	SP ↓ SI ↑	–	paste attributes ↓	Not changed	Reddy et al., (2015)
	potato starch	5, 10, 20 and 30 kGy	To, Tp, Tc ↓ ΔH ↓		SP ↓ SI ↑		paste viscosity ↓	cracks and pores appeared	Sujka et al., (2015)
	wild arrowhead	5, 10 and 15 kGy	To, Tp, Tc ↓ ΔH ↓	A type RC ↓ with dosage	SP ↓ SI ↑	–	paste attributes ↓	Not changed	A. A. Wani et al., (2015)
	buckwheat	0, 5, 10, 15 and 20 kGy			SP ↓ SI ↑				R. Verma et al., (2018)
	potato	0, 5, 10, 15 and 20 kGy			SP ↓ SI ↑				R. Verma et al., (2018)
	lotus stem	5, 10 and 20 kGy		B type RC ↓ with dosage	SP ↓ SI ↑		paste attributes ↓	granules destroyed at 20 kGy	Gani et al., (2013)
	red and white potato	0, 5, 10 and 20 kGy		B type RC ↓ with dosage	SP ↓ SI ↑		Pasting properties ↓	surface cracking appeared	Gani et al., (2014)
	chickpea starch	0, 4, 8 and 12 kGy		C type RC ↓ with dosage	SP ↓ SI ↑		Pasting viscosities ↓	granules cracked and clumped	Bashir & Aggarwal, (2017)
	high amylose maize	0, 30, and 60 kGy	To, Tp, Tc ↓ ΔH ↓	RC ↓ with dosage			Pasting viscosities ↓		Ocloo et al., (2016)
	native and acetylated wheat starch	1, 3, 5, 7, and 9 kGy	To, Tp, Tc ↓ ΔH ↓	A type RC ↑ with dosage			Pasting viscosities ↓		Kong et al., (2016)
		5, 10, 15, and 20 kGy	To, Tp, Tc ↑ ΔH ↓		SP ↓ SI ↑		Pasting properties ↓	ridges on the surface appeared	Mukhtar et al., (2017)
	lotus seed	5, 10, 15 and 20 kGy	To, Tp, Tc ↓ ΔH not changed	A type RC ↑ with dosage	SP ↓ SI ↑		Pasting properties ↓	roughness of surface at 20 kGy	Punia et al., (2020)
	arrowroot starch	0–15 kGy	To, Tp, Tc ↑ ΔH ↑				Pasting viscosities ↓	only a ↓ in granule size	Barroso & del Mastro, (2019)
	corn starch	3, 5, 10, 20 and 50 kGy	To, Tp, Tc ↓ ΔH ↓	No change in A pattern and RC			Pasting viscosities ↓	Not changed	Ben Bettaïeb et al., (2014)
	corn, potato and sour cassava	0–15 kGy		Slight ↓ in RC No change in A pattern				Granule size ↓ No change in surface	Teixeira et al., (2018)
	mung bean	0–5 kGy		No change in C pattern and RC	SP ↓ SI ↑		Pasting viscosities ↓	Few pores appeared	Castanha et al., (2019)
	tapioca starch	0, 5, 10 and 20 kGy	To, Tp, Tc ↑ ΔH ↓		SP ↓ SI ↑				Kanatt, (2020)
	rice starch	1, 2 and 5 kGy	To, Tp, Tc ↑ no change in ΔH	No change in A pattern and RC				No change in surface and size	Polesi et al., (2016)
	rice starch	1, 2 and 5 kGy			SP ↓ SI ↑	TDF not changed	Pasting properties ↓		Polesi et al., (2018)
Electron beam	rice starch	0–10 kGy	To, Tp, Tc ↓ ΔH ↓	A type RC ↓ with dosage		RS ↑, SDS ↓	Pasting properties ↓	Destruction of granules	Pan et al., (2020)
	potato	2, 4, 6, 8 and 10 kGy	To, Tp, Tc ↑ ΔH ↓	B type RC ↓ with dosage			Pasting viscosities ↓		Tappiban et al., (2022)
	corn	2, 4, 6, 8 and 10 kGy	To, Tp, Tc ↓ ΔH ↓	A type RC ↓ with dosage			Pasting viscosities ↓		Tappiban et al., (2022)
	rice	0–5 kGy		A type RC ↓ with dosage			Pasting viscosities ↓	No change was observed	Luo et al., (2019)
	waxy maize starch	2–30 kGy	To, Tp, Tc ↓ ΔH ↓	A type RC ↓ with dosage	SP ↓ SI ↑			No change was observed	Zhou et al., (2020)

### 3.2 Impact of ionizing radiation on techno-functional attributes of starch

#### 3.2.1 Morphology

Being a larger molecule than the other components of the food matrix, starch is particularly vulnerable to structural breakdown caused by ionizing irradiation. The starch origin, extent of irradiation (i.e. dose and time), moisture content of the specimen and the crystalline pattern of the starch are the dominant factors determining the morphological behavior of starch upon irradiation. In a recent attempt (Sudheesh et al., 2019) revealed the appearance of large fissures and cracks on kithul starch granules after irradiation which was accompanied by granules fragmentation at the highest level of gamma irradiation (10 kGy) (Fig. 2**c-f**). Comparable morphological changes were obtained in case of gamma irradiated broad bean starch (Sofi et al., 2013), bean starch (Gani et al., 2012, Hussain et al., 2014), lotus stem starch (Gani et al., 2013), red potato starch (Fig. 2**g-j**) (Gani et al., 2014) and lotus seed starch (Punia et al., 2020). On the contrary, there are also various reports indicating the insignificant impact of radiation on surface characteristics of starch like the study of (Teixeira et al., 2018) that studied the impact of 15 kGy gamma irradiation on the morphological attributes of potato, sour cassava and corn starches (Teixeira et al., 2018), or the (I. A. Wani et al., 2014) report which investigated the impact of 5, 10 and 15 kGy on the morphology of Indian horse chest nut starch.

Similar morphological transformations have also been reported for EB treated starches. For instance (Braşoveanu et al., 2013) showed the perforation of starch granules upon EB irradiation at 50 kGy (Fig. 2**b**). (Xue et al., 2017) also observed the creation of cracks and holes on corn starch granules during their irradiation with low doses of EB (<4 kGy). These results indicates how starches from different sources could exhibit different behavior upon treatment with ionizing radiation.

#### 3.2.2 Crystallinity

Starches with different crsytalline pattern have experienced various alterations upon irradition. In general, without any change in the crystalline pattern of the starch, its relative crystallinity would be reduced following irradiation which causes a drop in intensity of X-ray diffraction peaks. This change could be the result of the disordered starch granule’s double helices and the destruction of the ordered structure (i.e. amylopectin). For instance, (Sofi et al., 2013) obtained a remarkable decrease in the peak intensity of broad bean starches treated with 5–15 kGy gamma rays. Moreover the observed the disappearance of some characteristic peaks upon irradiation indicating the complete disruption of crystalline regions at specific sites within the granules. Similar findings were reported for gamma irradiated talipot palm starch (Navaf et al., 2022), lotus seed starch (Punia et al., 2020), oat and buckwheat starch (Dar et al., 2018), Indian Horse Chestnut starch (I. A. Wani et al., 2014), rice starch (Ashwar et al., 2014), brown rice starch (Kumar et al., 2017), and potato starch (Gani et al., 2014). However, in a more recent attempt, (Bashir & Aggarwal, 2017) observed the complete disappearance of characteristic picks of chickpea diffractogram upon gamma irradiation at 15 kGy (Fig. 2**k**) which could be the result of its more sensitivity against irradiation.

#### 3.2.3 Thermal properties

The thermal characteristics of oat starch and buckwheat were examined by (Dar et al., 2018) in relation to the gamma radiation effects (5, 10, 15, and 20 kGy). As the dosage rose from 5 to 20 kGy, they observed that the enthalpy and gelatinization temperature significantly dropped. (K. Verma et al., 2019) for cowpea & potato, (Kumar et al., 2017) for brown rice, (Bashir & Aggarwal, 2017) for chickpea starch, and (Punia et al., 2020) for lotus seed starch have all revealed similar findings. However, there is a discrepancy regarding the impact of irradiation on thermal transition temperatures (Onset gelatinization temperature (To), peak temperature (Tp) and conclusion temperature (Tc)). For instance, thermal transition temperatures of sago starch were remarkably elevated upon irradiation with 6–25 kGy gamma rays due to the generation of monosaccharides, oligosaccharides and short chain polysaccharides which delay the onset of gelatinization (Fig. 2**l**) (Othman et al., 2015). Similar increase in thermal transition temperatures of gamma irradiated oat starch was reported by (Mukhtar et al., 2017). On the contrary, (Punia et al., 2020), (Navaf et al., 2022), and (Bashir & Aggarwal, 2017) obtained a decline in thermal gelatinization temperatures of lotus seed, talipot and chickpea starches, respectively, which are mainly caused by the weakening/destruction of ordered/crystalline structures within the granule following irradiation.

#### 3.2.4 Swelling power and solubility

When exposed to ionizing radiations up to 20 kGy, the starches' water absorption capacity (WAC) increases. The structural unwinding of starch branches and the development of simple sugars with a stronger affinity for water are considered to be the causes of the increased WAC. Because it affects various functional and sensory aspects of the food, water absorption is essential to the food preparation process (Verma et al., 2018). The swelling capacity of starch-based foods is significantly increased by irradiation, often up to 5 kGy and at temperatures below 80 °C. Beyond 5 kGy, however, a considerable reduction in the swelling power is seen because of severe structural defragmentation (Jagannadham et al., 2014). On the other hand, the starch solubility rises with irradiation dosage due to the structural breakdown of polymers. The depolymerization of starch molecules (i.e. amylopectin branches and amylose), and proteins caused by radiation and the emergence of more soluble simple sugars are thought to be the causes of the rise in the solubility index (Dar et al., 2018; Bashir et al., 2017; Jagannadham, 2014).

#### 3.2.5 Rheological and pasting characteristics

In general, the structural breakdown of starch molecules caused by irradiation, lowers the pasting characteristics (e.g. trough, peak, final, and breakdown viscosities), and rheological features (e.g. consistency index, and flow behavior index). The setback viscosity values provide details on starch retrogradation and syneresis during thawing and storage. The primary chemical that causes greater levels of retrogradation and ultimate viscosity is amylose. Pasting characteristics can be simply linked to the textural characteristics and the final product quality; peak viscosity provides an idea of the capacity to bind water, while a higher breakdown value indicates that the final product will taste better. These characteristics are quite significant and helpful when developing the processing parameters (Verma et al., 2019; Dar, 2018; Bashir et al., 2017).

Singh et al. (2011) observed that irradiation (0.01, 0.05, 0.1, and 0.5 kGy) significantly changed the rheological characteristics of potato starches. Trough, peak, final, breakdown viscosities, and gel hardness all showed a tendency to decrease with increasing irradiation dosage. But when the irradiation dosage increased, a rise in gel cohesiveness was seen. With an elevated dosage, a noticeable decline in loss moduli and storage was seen. Similar results were found for cowpea, potato (Verma, 2019); kithul (Sudheesh, 2019); oat, buckwheat (Fig. 2m) (Dar et al., 2018); brown rice (Kumar, 2017); sago (Othman et al., 2015); and cowpea starches (Abu et al., 2006).

## 4 Impacts of non-ionizing radiations on starch

### 4.1 Microwave (MW) irradiation

Microwaves are non-ionizing radiation that has frequencies between infrared and radio waves (300 MHz-30 GHz). Microwave is electromagnetic waves of radiant energy (Demirkesen et al., 2011). During MW treatment, oscillating microwaves penetrate the food matrix and causes a vibration that generates heat through friction. The polar molecules play the main role in this phenomenon where they try to align themselves in the direction of the applied field. Millions of times every second, the microwave field reverses polarity. A significant amount of kinetic energy is generated from the oscillating electric field by the dipoles and is transmitted to other molecules by the collisions. MW treatment is an effective procedure with high time efficiency that generate internal heat and transfer it through conduction, convection, and evaporation (Wang et al., 2021).

#### 4.1.1 Impact of microwave irradiation on starch

##### 4.1.1.1 Chemical composition

Microwave radiation modifies the physicochemical characteristics of starch by affecting water evaporation, swelling power, crude protein, and solubility. These characteristics can vary depending on the type of starch, moisture content, processing time, temperature, and absorbed energy. Numerous investigations have employed microwave treatment to modify starch characteristics. It has been reported that microwave could reduce moisture content, crude protein, crude fiber, water absorption, sugar level and absolute density while it increased the ash level (Rostamabadi et al., 2022). However, water-soluble minerals may occasionally diffuse from starch into water after microwave treatment, resulting in relatively little mineral loss and a decrease in the amount of ash. The digestibility, morphology and crystal structure of the granules may also alter as a result of this treatment. Studies in the past suggested that MW treatment could enhance resistant starches (Zailani et al., 2022). Additionally, it results in the breakage of starch's lengthy dextran chains.

In the case of starch, the molecular rearrangements, which alter the hydration and pasting properties, require a little amount of water in order to absorb radiation and to have a plasticizing impact. Thus, the moisture content is one of the main factors that influences the extent of alterations in presence of irradiation. In an attempt, the ability of MW to alter the techno-functional characteristics and gel viscoelasticity of starchy flour system was investigated by Solaesa et al. (2021). The low moisture content of the remaining samples prevented granular disruption, but the rice flours treated at 20% and 30% moisture content were pregelatinized.

MW irradiation may cause a break in α-1,4 and α-1,6 glycosidic linkages. Thus, the amylose and amylopectin content could be affected upon MW. It has been found that the amylose content of MW treated starches is significantly higher than the native starch, and this can be predicted by the disintegration of amylopectin branches and its long-chain structure. Moreover, the amylose that was held inside the granule structure by the amylopectin groups can escape the semi-crystalline structure due to the swelling of starch during MW and contribute to the measured amylose content (Demirkesen et al., 2014). It has been reported that waxy starches are more vulnerable to degradation compared to amylose containing starches that indicates the resistibility of amylose against irradiation (Zailani et al., 2022).

##### 4.1.1.2 Chain length/distribution and molecular weight

As the α-1,6-glycosidic links in the internal chains (amorphous lamellae) are less hampered by the steric hindrance and are more mobile than those located in the crystalline lamellae, they appeared to be more vulnerable to MW energy, which is the likely the source of a selective degradation. It has been stated that the central amorphous region is more susceptible to degradation. The major degradation occurs in the internal chains at the first stage (5 min), while the external chains (crystalline region) mostly destroy at the second stage (10 min) as stated by (Yang et al., 2017). They also corroborated the breakdown of starch molecules during MW irradiation, which resulted in a drop in chain length distribution and molecular weight of waxy maize starch. The results of ^1^HNMR spectroscopy further supported these conclusions (Yang et al., 2017). This finding is in agreement with the study of Wang et al. (2021) in which degradation of internal chains was related to the stronger MW energy conversion capacity. Chen et al. (2021) suggested that the chain length distribution of both A chains and B chains had no significant difference, but the molecular sizes were obviously decreased after MW treatment. Thus, the authors suggested that the continuous MW treatment might unravel the double helical structures and cut the central C chains, not the external branches of amylopectin. In order to understand the role of MW treatment on starch, Zhong et al. (2021) evaluated the structural changes of amylose and amylopectin under MW irradiation. It has been suggested that both amylose and amylopectin granules are differently sensitive to MWs. Microwave treatment, especially during the first 3 min, decreased the molecular weight of amylose and amylopectin molecules (Table 2).

**Table 2:** An overview on the effect of non-ionizing radiations on physicochemical and techno-functional attributes of different starches.

**Type of radiation**	**Starch type**	**Radiation condition**	**Assessed attributes**	**Key findings**	**Reference**
Microwave	Native waxy maize starch	1600 W − 5 min	Chain length, Molecular weight	• No change in crystalline regions (A-chain) with short MW time (5 min) ↓ A-chain content of maize starch with increasing MW time. ↓ Molecular weight with ↑ MW time	Yang et al. (2017)
	Lotus seed starch-chlorogenic acid mixtures	300 W, 2450 MHz for 8 min	Chain length, The water holding capacity, Stability	• ↑ Cleavage and release of internal amylopectin chains with MW treatment ↑ Cleavage of internal B2 and B3 amylopectin chains Cleavage of internal B2 and B3 amylopectin chains was greater upon MW Chlorogenic acid lowered retrogradability of starch after MW treatment	Wang et al. (2021)
	Hull-less barley starch	640, 720, 800 W for 60, 120 and 180 s	Chain length Molecular size	• No significant difference in chain length distribution of both A chains and B chains ↑ Cutting C chains ↓ Molecular sizes	Chen et al. (2021)
	Elephant foot yam starch	520 W for 120 s, at 35% moisture content	Morphology, Amylose content, molecular weight, Rhology, Thermal properties, crystallinity	• ↑ Surface roughness with MW ↓ Molecular weight with MW ↑ Amylose with MW ↓ Molecular weight with MW ↑ Gelatinization temperatures but ↓ΔH ↑ G′ and G′′ moduli → a strong gel network under high shear. Surface degradation and particle aggregations with MW Greater extent of short amylopectin compartment after MW. Reduction in relative crystallinity and enthalpy upon MW.	Barua et al. (2022)
	Pure amylose & amylopectin starch	2450 MHz, 800 W for 1, 2, 3, 4, 6, and 8 min	Chain length Molecular size	• For amylopectin: ↓ DP > 16 → ↑ DP < 16 especially↑ DP 11 after 1 min No more significant change with further treatment Narrower chain length distribution in pure amylose starch granules a preferential degradation of long side chains. For amylose; ↑ DP < 10, ↓ Molecular weight	Zhong et al. (2021)
	Sago Starch	900 W, for 15 min	Ash, amylose, resistant starch content, oil and water binding capacity, morphology	• ↑ Ash content ↑ Amylose content ↑ Oil and water binding capacity ↑Resistant starch with MW Surface degradation and particle aggregations with MW Increase in apparent amylose content greater oil binding capacity by the MW treated starches associated to fissures on granules surface Modified starches possessed greater RS content after cooking ↑Solubility and ↑swelling powers at lower temperature but ↓ Solubility and ↓ swelling powers at higher temperature ↓ Paste clarity with MW treatment	Zailani et al. (2022)
	native taro starch and starch nanoparticles	450 W for 5 min	Morphology, crystallinity	• ↓ The sizes of the starch granules Most of the particles turned into starch nanoparticles. ↓ Crystallinity in MW treated starches/starch nanoparticles	Das and Sit (2021)
	Lotus Seed Starch	2.4, 4.0, 6.4, or 8.0 W/g	Swelling power, amylose content, digestible and resistant starch, Crystallinity, molecular interactions	• ↓ The swelling power, ↓ amylose leaching with MW treatment ↑ the resistant starch, ↑slowly digestible starch, ↓glycemic index with MW treatment Due to the change in distribution of water within granules, new starch–water interactions were formed. New double helical structures formed and crystallinity enhanced The SP and solubility and starch digestibility reduced upon MW	Zeng et al. (2016)
	Waxy and non-waxy hull-less barley starch	640 W, 720 W, and 800 W FOR 60 s, 120 s, and 180 s	Thermal properties, Pasting/Rheology, Molecular structure	• MW ↑ gelatinization temperatures in non– waxy starch MW ↑ gelatinization temperatures in waxy starch ↑ MW → ↓Relative crystallinity ↓ΔH of in-kernel starches ↑ MW → ↑ Relative crystallinity ↑ ΔH of isolated starches ↓ viscosities of in-kernel starches with ↑ MW power and time No change viscosities of isolated starches For MW treated in-kernel starch: within the cells the crystalline regions was disrupted thereby reduced the ratio of 1047/1022 cm^−1^ wavelengths, ΔH and relative crystallinity For MW treated isolated starch: disruption of amorphous structure and increase in the amount of remaining double helix structure accompanied by a decrease in the ratio of 1047/1022 cm^− 1^ wavelengths and an increase in ΔH of isolated starch	Ma et al. (2020)
	Pure amylose and amylopectin starch	2450 MHz, 800 W and 1, 2, 3, 4, 6, and 8 min	Rheology	• ↑ G′ and G′′ moduli (viscoelastic behaviour) with shorter (0–3 min) mirowave treatment ↓G′ and G′′ moduli (viscoelastic behaviour) with longer (3–8 min) microwave treatment High amylose degradation/aggregation and formation of V-type crystalline, and ↑ crystallinity for the first 2 min ↓ resistant starch content → amylose re-aggregation without involving amylopectin Upon prolonged treatment of amylose granules → the resistant starch ↑ and water solubility ↑ Initially, the crystallinity of amylose starch ↑ & The crystallinity of amylopectin ↓ but with further treatment; the crystallinity of amylose starch ↓ & The crystallinity of amylopectin ↑	Zhong et al. (2021)
	Hull-less barley starch	2450 MHz, 800 W and 1, 2, 3, 4, 6, and 8 min	Rheology	• Microwave irradiation of in-kernel starches ↑ more amylose breakdown than that of isolated starches ↓ smaller K values. MW → The stability of the molecular structure ↓ TGʹmax of starch pastes. ↑ Gʹmax and Gʹ90°C of most in-kernel MW isolated hull-less barley kernel starch and isolated MW isolated hull-less barley kernel starch Strength, gel, and Gʹ25°C, Gʹ0.1 Hz and Gʹ20Hz in in-kernel MW isolated hull-less barley kernel starch > untreated starches. But no significant changes in chain-length distributions of A and B chains	Chen et al. (2021)
	High-amylose maize starch	2450 MHz, 1.2 kW for 1–4 min	Digestibility, viscosity, crystallinity	• Partial amylopectin and amylose broken down in 1 min treatmentViscosity diminished and RS (RS) enhanced upon 1 min treatment. Crystalline lamella reordered in 2–4 min MW treatment. Viscosity enhanced and RS diminished in 2–4 min MW treatment.	Zhong et al., (2019)
	Cassava starch	600 and 700 W for 0, 5, 15, 30 and 60 s	Crystallinity, thermal attributes, rheology	• Crystalline pattern was not changed. Gelatinization temperature increased but SP water absorption capacity and RC diminished. Peak and set back viscosity reduced with MW treatment but pasting temperature elevated.	Oyeyinka et al., (2021)
	Rice starch	540 W for 0, 10, 20 and 30 min	Thermal attributes, rheology	• MW treatment provoked minor change in starch, but drastically changed the behavior of starch upon further annealing modification.By untangling the entanglements, MW simplified the rearrangement of starch molecules through subsequent annealing process.	Zhong et al., (2020)
	Rice starch	800 W or 1600 W for 1–5 min	Radical generation	• MW power and moisture content have impact on the extent and type of radical created. Generated radials were stable and existed for a long time at room temperature. The main radical components were those located on C1 position of glucose ring.	Fan et al., (2016)
	high amylose corn starch	microwave time (2–4 min) and power (20–100%)	Digestibility Physicochemical attributes	• The RS contents of the samples increased with increasing microwave-storing cycle The solubility, WBC and RVA viscosity values of the microwave treated samples were higher than those of native starches	(Mutlu et al., 2017)
	Maiz flour (starch)	400 W for 0.5, 1, 2 and 4 min	Crystallinity, thermal properties, viscosity	• SEM micrographs proved more swollen starch granules following MW treatment MW induced the generation of V-type allomorphs Gelatinization temperatures and ΔH elevated upon MW Sever MW conditions reduced starch viscosity	Román et al., (2015)
				
Radio frequency	potato starch	6 kW, 27.12 MHz for 5, 10, 15, 20 and 30 mins	morphology, amylose content, microstructure, viscosity, and thermal properties	• RF treatment roughened the starch surface RF treatment declined amylose content, viscosity and pasting temperature of the starch RF-treated starch gels possessed superior printability RF treatment rearrange the internal architecture of the starch granules, making them more ordered and stable.	Ma et al., (2022)
	rice starch	6 kW, 27.12 MHz for 10, 15, and 20 min	structural, digestive and physicochemical properties	• RF treatment enhanced the RS content and reduced the RDS content of starches granular surface erosions developed upon RF Applying RF did not change the A-type crystalline pattern but diminished RC and double helix components. peak viscosity and breakdown of indica and japonica rice starch diminished upon RF while waxy starch revealed higher values for these parameters after RF. Thermal attributes showed an increase in granular stability upon RF.	(Zhang et al., 2022)
	potato starch	27 MHz for 5 min	morphology, amylose content, microstructure, viscosity and thermal properties	• MW treatment was more potent in reducing the amylose content and changing the granule surface. Granule size of RF treated starch was smaller than MW treated ones. RF treatment changed the crystalline architecture and diminished the RC of the starch, but the MW treatment implied no effect. Gelatinization temperatures diminished following RF treatment. peak and the breakdown viscosity of RF treated samples was remarkably higher than that of MW treated ones.	Xia et al., (2018)
	rice flour	6 kW, 27.12 MHz for 5, 10, and 15 min	SEM, RVA, DSC, XRD	• RF treatment provoked aggregation of starch granules and enhanced their size.RF treatment induced a reduction in RC and short-range order of granules.RS was greater in RF modified starches. The G’ and G’’ of RF treated samples were increased while reverse trend was observed in terms of tan δ	Zhang et al. (2022)
Ultraviolet (UV)	corn, waxy corn, wheat and potato	wavelength: 254 nm; intensity: 33.4 W/m2 for 8 h	XRD, HPSEC, physicochemical properties	• Moisture content and RC diminished upon UV irradiation. UV induced a reduction in molecular weight of corn, waxy corn and wheat while it was vice versa in case of potato starch. Potato starch molecules showed aggregation due to recombination of radicalized molecules. Potato possessed the least stability against UV irradiation.	Bajer et al., (2013)
	cassava starch	wavelength: 256 nm for 1 h	RVA, DSC, XRD	• setback and final viscosities were diminished after UV irradiation. RC and ΔH were reduced upon UV treatment The surface of starch granules were rougher after UV treatment. However, A-type crystalline pattern stayed intact. The a* value was lower in UV modified starches.	Hornung et al., (2016)
	Wheat starch	UV-C wavelength of 254 nm and UV-A wavelength of nm for 1,6,12 and 24 h	physicochemical properties, RVA, DSC, XRD, FTIR	• Radiation up to 12 h generates new bounds among starch chains but destroyed them after 12 h irradiation. transparency of formed films diminished upon UV treatment due to the formation of new interaction among molecules. The surface of generated films were the same for both types of UV applied. The reduced intrinsic viscosity if modified starch confirmed the breakage of molecules into smaller fractions.	Shahabi-Ghahfarrokhi et al., (2019)
	cassava starch	wavelength: 256 nm for 1 h	physicochemical properties, RVA, DSC, XRD, FTIR	• TGA curves were identical for all native and modified samples. Peak viscosity, enthalpy of gelatinization, relative crystallinity of starch were decreased after UV modification. The surface of the modified starches were rougher. Modified starches were whiter on surface possessing a greater L* value.	Hornung et al., (2015)
Infrared	Indian Horse Chestnut starch	for 15 s, 30 s, & 45 s	physicochemical properties, SEM RVA, DSC, XRD, FTIR	• Radiation caused surface cracks.Water absorption capacity and light transmittance were improved while apparent amylose content, pH and syneresis diminished upon IR treatment.IR modification reduced RVA viscosities of starch. gelatinization temperatures and ΔH were lower in modified starches. Modified starches possessed greater antioxidant activity.	Shah et al., (2016)

##### 4.1.1.3 Morphology

**Fig. 3:**
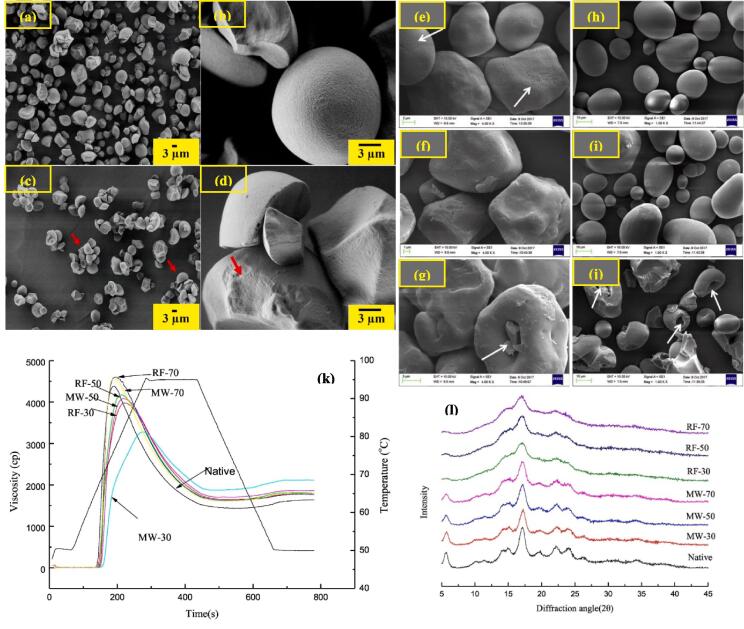
SEM micrographs of native (a and b) a nd MW treated (c and d) elephant foot yam starches, reprinted with permission from (Barua et al. 2022); SEM micrographs of native maize starch (e), maize starch MW treated at low (f) and high (g) power, and native potato starch (h), potato starch MW treated at low (i) and high power (j), reprinted with permission from (Xu et al., 2019); RVA plot (k) and X-ray diffractogram (l) of potato starch treated with MW and radiofrequency (RF) at different starch concentrations (30, 50 and 70), reprinted with permission from (Xia et al., 2018).

The morphological properties of starch are mostly dependent on the amount of applied MW treatment where some of the starch granules can retain their structure when treatment is below the gelatinization temperature while, beyond the gelatinization temperature the granular structure could be completely destroyed (Mhaske et al., 2021). During MW treatment, collision and friction between polar water molecules may cause the surface damages resulting in formation aggregates or clusters(Fig. 3**c)** (Barua et al. 2022). Pinholes, fissures, and fractures are also observed on MW heated starch granules (Fig. 3**d)** (Barua et al., 2022, Zailani et al., 2022). The surface degradation and particle aggregations could be ascribed to the collision of polar water molecules to each other upon MW treatment. Besides, the expansion of granules during water vaporization might increase the particle size resulting in the development of multiple aggregates (Barua et al. 2022). The sago starch granules treated by MW treatment had a rough surface as opposed to the smooth surface of the native and starch granules treated with cold-soaking. Furthermore, due to the greater amylose content of the modified starches, excessive amylose leaching caused the starch granules to agglomerate (Zailani et al., 2022). Similar changes was also reported by Xu et al., (2019) for MW treated corn (Fig. 3**e-g**) and potato (Fig. 3**h-j**) starches.

##### 4.1.1.4 Swelling, solubility and paste viscosity

Both swelling power and solubility provide information about the degree of interactions between starch chains within the amorphous and crystalline domains of the granule. Microwave heating decreases swelling power due to the improved formation of inter/intra-molecular bonding through amylose-amylose, amylopectin-amylopectin, amylose-amylopectin interactions and amylose–lipid complexes (Mhaske et al., 2021). The appearance of new fluorescent spots in the interior of the granules through confocal laser scanning microscopy corroborated that a high microwave power treatment damaged the granular structure of starch, making it easier for starch chains that had previously been buried in the granules to be exposed and providing additional binding sites (Wang et al., 2019). The partially degraded amylose and amylopectin chains in starch molecules lead to an increase in the number of short chains during the MW process which decreases the swelling power of starch. Another reason for decreased swelling power and viscosity of starch may be attributed to the restructuring that occurs inside granules. When starch granules are gradually heated in excess water, the starch granules start to swell, resulting in the leaching of the amylose molecules into the supernatant. This causes the formation of a network surrounding the swollen granules, which inhibits further swelling of starch. Reportedly, at the end of gelatinization, the amylose leaching decreases with increasing MW power (Zeng et al., 2016). In a recent instance, (Y. Li et al., 2019) studied the swelling and pasting behavior of MW treated millet starch. They outlined that the swelling power and past viscosity of starch was remarkably declined following MW treatment. This impact was even more noticeable at greater levels of moisture content and was mostly relevant to the physical damage and disintegration of starch granules upon MW irradiation. Lower swelling power and pasting viscosities were also observed in MW treated lentil starch (González and Pérez, 2002) and potato starch (Xia et al., 2018) (Fig. 3**k**). The swelling power values of waxy and non-waxy hull-less barley kernels reduced with the increasing MW conditions (power × time). The internal structure of starch granules varies as a result of MW radiation, which may result in a rearrangement in the crystalline areas and cause variations in starch swelling (Ma et al., 2020). A drastic decrease in the swelling power of MW treated starch was observed at high temperatures (90 °C) and ascribed to the irreversible rupture of granules at this temperature (Zailani et al., 2022). The effects of amylose and amylopectin on swelling power were examined independently by (Zhong et al., 2021). In comparison to amylopectin, amylose was shown to have the least swelling power. Indeed, the network that forms around the enlarged granules created by leached amylose molecules during heating prevents additional swelling. In another attempt, Zhong et al., (2021) studied the solubility of MW treated starches. They outlined that MW treatment through the production of soluble chains (low molecular weight fractions) could enhance the solubility of starch molecules.

##### 4.1.1.5 Thermal attributes

Microwave electromagnetic energy, when used in the optimal process conditions, encourages rapid heating of starch granules while accelerating disentanglement and local branch breaking of amylopectin. In the study of Barua et al. (2022), MW energy resulted in the production of short chain amylose crystals, which led to the formation of two sharp endotherm peaks on DSC thermogram. The changes in starch structure, such as interactions between amylose and amylose, inhibit the movement of its chains. This could provoke a rise in T_0_, T_P_, and T_C_ and ΔH. However, in case of MW treated elephant foot yam starches the breakage of the hydrogen bonds that connect the double helices increased the helical arrangement's mobility and lowered the total amount of ΔH (Barua et al., 2022). They stated that the local breaking of amylopectin branches upon MW treatment increased the development of short chain amylose, which lowered ΔH. Therefore, during MW treatment, the development of disorderness was mainly accompanied by an increase in short chain amylose fractions. Nonetheless, a variety of thermal behaviors have been reported for different starches. For example, starches of C type have lower T_o_ and T_p_ than A type starches. This is attributed to the unique arrangement of A and B type polymorphs in the C type starch granules. The B type polymorphs are located in the center of starch granules, which are surrounded by the A type polymorphs. During gelatinization, the melting of the crystalline structure is initiated from the central hilum of granules since the B type polymorphs possess a lower melting temperature than the A type polymorphs because of their looser packing. Moreover, starches having the longer branch chains of amylopectin/intermediate components and the high amylose content form thermally more stable and long-chain double-helical crystallites, resulting in higher T_p_ and T_c_ (Ambigaipalan et al., 2014).

##### 4.1.1.6 Fourier transform infrared spectroscopy (FTIR) studies

FTIR may also give information about the molecular structure of starches. The ratio of 1047/1023 and 995/1023, can be used to assess the internal changes in the ordered compartments, which refers to the relative crystallinity. In a very recent attempt, Zailani et al., (2022) stated that MW treated sago starch possessed lower 1047/1023 ratios than the native starch, but they exhibited a greater 995/1023 ratio than the native starch. The lower 1047/1023 ratios of modified starches imply that they may contain lower ordered fractions. This may have happened because the ordered crystalline structure was disturbed by the partial gelatinization that took place upon MW. On the other hand, the leaching of amylose during MW treatment enhances the interactions among leached molecules through hydrogen bondings. Hence, a higher 995/1023 ratio was displayed by the modified starches, showing a higher proportion of double helix amylose (Zailani et al., 2022). Ma et al (2020) observed that MW decreased the ratio of 1047/1022 cm^−1^ wavelengths, ΔH and relative crystallinity of in-kernel waxy barley starch. This corroborated the disruption of crystalline regions within the cells. In the case of isolated starch, however, MW decreased the ratio of 1047/1022 cm^− 1^ wavelengths but elevated the ΔH. The enhance in ΔH might be related to the disruption of amorphous structure and an increase in the amount of remaining double helix structure with MW treatment.

##### 4.1.1.7 Rheological properties

The changes in starch granular structure and size through MW might affect its rheological properties. As an instance, Zhong et al. (2021) stated that the rheological properties of pure granular amylopectin (waxy) starch were not changed by a mild (1–2 min) MW treatment. However, at severe conditions, the molecular degradation of amylopectin molecules by MW irradiation reduced the gel strength, but elevated the plastic behavior and deformability. On the other hand, the viscoelastic behavior and starch gel strength were intensified by a mild MW treatment. In another recent work, Chen and his collogues (2021) studied the impact of MW irradiation on rheological behavior of hull-less barley starch. They outlined that the viscosity of starch could be remarkably diminished upon MW treatment where irradiated starch displayed a less thickening more thinning behavior. They further investigated the molecular structure of starch molecules and revealed that such variations in rheology could be caused by the unraveling of double helix structures and degradation of amylopectin molecules (Chen et al., 2021). In the study of Barua et al. (2022), the dynamic rheological assessments revealed an enhance in viscoelastic properties. This might be related to the fact that the higher presence of shorter amylose chains with greater mobility could favor the lateral interchain interactions through their facile re-alignment, thereby creating more double helical structures and coarse aggregates.

##### 4.1.1.8 Crystallinity

X-ray spectra of MW irradiated starch has revealed that, upon relatively short irradiation times, the relative crystallinity of the treated samples almost remains unaltered (Yang et al., 2017). This implies that the amorphous region had been more prone to destruction during microwave radiation. In an interesting study, (Han et al., 2021) studied the structural variation of rice starch during constant power microwave (CPM) treatment. Through small and wide angle XRD assessments they obtained a remarkable decrease in RC and double helix content of granules upon CPM treatment. Indeed, water is the main constituent of starch receiving the microwave radiations. Water molecules start extensive micromovements and finally evaporate within the granules which generate extremely great internal vapor pressures resulting in the degradation of starch molecules at amorphous/crystalline regions. Yang et al., (2017) investigated the structural transformation of MW irradiated waxy maize starch and corroborated that the intensity of various XRD peaks was dramatically reduced and the relative crystallinity dropped when the irradiation duration was extended from 5 min to 10 and 20 min, showing that microwave irradiation could affect the entire crystalline area at more severe processing conditions. Similar results has been reported for MW irradiated Lotus seed starch (Nawaz et al., 2018), potato starch (Fig. 3**k**) (Xia et al., 2018, Xu et al., 2019), and millet starch (Y. Li et al., 2019).

### 4.2 Other non-ionizing radiations

Apart from microwave, other types of non-ionizing radiations viz. radiofrequency radiation, ultraviolet radiation as well as infrared radiation could also provoke various alterations to physicochemical, structural and nutritional attributes of starches. However, compared with microwave, limited studies have investigated the impact of these methods on starch characteristics. Radiofrequency radiations are electromagnetic waves possessing the longest wavelengths and smallest frequencies (1––300 MHz) which induce severe molecular rotation/frictions within the medium. Although the principle of generating heat upon RF is similar to the microwave process, the product processed by RF possesses superior quality. Given the longer wavelength of RF waves, they are more potent to penetrate deep into the medium thereby distributing heat more uniformly throughout the product. Hitherto, the successful application of RF in food processing has been reported by numerous researchers (Zhang et al., 2018). RF treatment was also found to be an efficient tool to provoke various changes in structural, physicochemical and digestibility of different starches. As an instance, Xia and his colleagues, (2018), studied various characteristics of potato starch modified via RF (27 MHz for 5 min) and MW (2450 MHz, 800 W for 5) treatments and outlined that starches treated with MW possessed rougher surface and lesser amylose content versus RF treated starches. MW treated starches had greater granule size than RF treated starches. Their results also showed that RF modified starches had superior peak and breakdown viscosity in comparison with MW treated ones (Fig. 3**k**). They also indicated although MW induced no change in starch crystallinity, RF diminished the RC of modified starches (Fig. 3**l**) and concluded that RF could be a potent technique for annealing modification of starches. The impact of RF on physicochemical and structural attributes of starch are provided in detail in Table 2.

Ultraviolet radiation is another type of electromagnetic radiation with wavelengths ranging from 100 to 400 nm. UV through indirect photochemical reactions, such as the generation of free radicals, might cause ultrastructural alterations. Reportedly, the surface, hydrophilicity, and thermal stability of polysaccharides can be altered upon UV radiation. Due to the poor penetration of UV radiation, rheological and textural changes could occur only at the surface of UV irradiated materials (Pandiselvam et al., 2022). In this line, in an attempt, (Bajer et al., 2013), studied the structure and attributes of corn, waxy corn, wheat and potato starches treated with UV radiation and stated that, generally, the changes in chemical structure of UV irradiated starches were small. However, moisture content and RC were marginally diminished. A decline in amylose content upon UV radiation was ascribed to its lower photostability than amylopectin and its localization at the periphery of the granules which elevate its expose irradiated UV. From the FRIT results, these researchers suggested the occurrence of some photochemical reactions including cross-linking or photolysis reactions where potato starch experienced the most changes. XRD results also corroborated the severe amorphization of amorphization of potato starch with 50% decrement in its crystallinity upon UV treatment. A reduction in molecular weight of irradiated starches was well reflected on their HPSEC plots which could be mainly due to scission of main chain of molecules through dehydroxylation, dehydrogenation and glycoside ring opening.

## 5 Applications of irradiated starches

Starch-based materials have received great attention in the agricultural, food, packaging, and biomedical sectors owing to their biodegradability and low cost (Bhuyan et al., 2016). Applying radiation is a promising approach to introduce novel features (i.e. degradation, crosslinking, and grafting) to the starch composed systems (Cieśla & Abramowska, 2021). This section and Table 3 represent the applications of irradiated starches in the realm of food.

**Table 3:** Food applications of irradiated starches.

**Starch**	**Radiation Type and condition**	**Application**	**Assessed attributes**	**Effects on the final product**	**Reference**
Corn, potato, and cassava	Gamma 0–15 kGy	Films (casting)	Colour and resistance to breakage	• Increasing the dose augmented the resistance to breakage in corn, and potato starch films. .In the case of cassava, resistance to breakege was diminised by irradiaion. Irradiation at 15 kGy adversly promoted the parameter b* (yellow color)	Teixeira et al., (2018)
Potato	Gamma 0 to 50 kGy.	Starch-PVA Blend Films (casting)	Tensile properties, TGA, dynamic mechanical analysis, and application on guava storage.	• The tensile strength increased with an increase in irradiation dose and attained a maximum value at 25 kGy. Radiation improved the thermal stability of starch-PVA blend films. Irradiation adversly made the blend films glassier. The film induced less weight loss during guava storage due to the crosslinking effect provoked by irradiation.	Dafader et al., (2017)
Plasticized corn starch (PLST) and Carboxymethyl cellulose	Gamma 10–50 kGy	Film (casting)	Thermal, tensile and mechanical properties, antibacterial activity	• The thermal stability, tensile strength, and elongation at break increased with an increasing irradiation dose. The gamma-irradiated blends presented higher antimicrobial properties than the unirradiated blends.	Senna et al., (2016)
Corn	Gamma 5, 10, 20, 30, 50 and 75 kGy	Film using Poly(vinyl alcohol) by casting	Tensile strength, mechanical properties, contact angle to the water, swelling behavior, and determination of gel fraction	• Tensile strength was not affected by irradiation performed with doses in a range of 5–75 kGy Doses of 20 kGy or higher decreased flexibility, and using doses from 30 kGy probably also decreases the elasticity of the material. A dose of 5 kGy decreased the surface hydrophilicity of the films and increased the irradiated material solubility.	Cieśla & Abramowska, (2021)
Potato	Gamma 5, 10, 15, 20, and 25 kGy	Acrilic-acid-grafited-starch hydrogel	Swelling and thermal properties and determination of dye adsorption	• The higher swelling capacity was found when 15 kGy dose applied.Radiation did not interfere with the decomposition temperature.At highest dose of irradiation, the swelling index of gels adversely diminished due to the hydrolysis of molecules. The hydrogels were efficient in adsorbing dye and can be applied to pharmaceutical, agricultural and environmental technology.	Bhuyan et al., (2016)
Cassava	Gamma 25.0, 50.0, and 100.0 kGy	Foam trays (extrusion)	Compression strength, flexibility, barrier properties, density, thickness, and aerobic biodegradation by mass loss	• At elevated irradiation doses, the foam compression strength and flexibility diminished The density decreased while the thickness increased with irradiation. Aerobic biodegradation time slightly increased for the samples containing irradiated starch, owing to small parcels of crosslinked polymer which slowed down their biodegradation.	Brant et al., (2018)
Corn	Gamma 10, 20, 30, and 40 kGy	Film	Tensile strength, Water vapor permeability, and crystallinity	• Irradiation doses increased the tensile strength and decreased water vapor permeability. Taking energy consumption into consideration, the authors recommended 30 kGy as the optimal irradiation dose for corn starch. The 30 kGy irradiated corn starch film showed great potential for developing biodegradable starch film with improved properties.	Li et al., (2018)
Waxy maize	Electron beam 2, 4, 6, 8, 10, 15, 20 and 30 kGy	Films (casting)	Chromaticity, mechanical properties, and solubility	• 10 kGy dose of electron beam irradiation moderately degrade starch and increase the number of linear chains, thus resulting in starch films with better tensile strength, elongation values, and enhanced solubility.At doses higher than 10 kGy, more α-1,4 bonds would be cleaved which could interefere with the film forming ability of the starch molecules.At doses below 15 kGy, films possessed lighter color.At doses more that 15 kGy, films were both darker and yellower (less L values) and more yellow (higher b value), indicating the careamelization of starch.	Zhou et al., (2020)
Carboxymethyl-sago	Electron beam 25, 30 and 35 kGy	Hydrogel	Relation between the degree of substitution of Carboxymethyl-sago starch and electron beam doses, swelling, and thermal properties	• There was a significant interaction between the degree of substitution of the Carboxymethyl-sago starch and irradiation doses on gel fraction and swelling ability of the hydrogel.	Zahib et al., (2021)
Rice	Electron beam 25 kGy	Copolymer hydrogelsStarch/poly(ethylene oxide) in different proportions	Reticulation efficiency Swelling behavior at different pH, thermal properties, morphology, and drug release	• Improve in reticulation and consequently the physical and chemical properties with irradiation. Irradiation imparted no change in thermal properties drug release of gels.	Zhou et al., (2020)

### 5.1 Formation of films and packaging from irradiated starch

Besides being cheap and efficient, the packing industry understands that synthetic polymer-based packaging are not biodegradable and cause environmental damage. For this reason, studies on starch and starch composites have been initiated in recent years to elaborate starch based films and packings. Normally, starch in its native form is not suitable for such applications, but when modified, can be employed alone or in blends/composites to improve the barrier properties and thermal and mechanical characteristics of the films (Liu et al., 2022, Nadia and Othman, 2019). Furthermore, studies using irradiated starch in association with other polymers can be an alternative to currently used synthetic polymeric materials (Dafader et al., 2017).

The commonly used biopolymers are starch, chitosan, alginate, gelatin, and shellac, but poor mechanical properties and hydrophilic nature are the major drawbacks of the films made with these materials. For this reason, numerous studies are in progress to overcome these limitations to offer physicochemical characteristics comparable to those of synthetic polymer-based materials (Dafader et al., 2017); within these studies is the use of radiation. The advantage of using radiation method is that processes induced by irradiation limit the use of strong chemicals, i.e., crosslinking agents. Therefore, the radiation can efficiently modify starch for future use in film and packaging applications or on ready films. The improvement of the properties of such systems was attributed to the crosslinking/grafting processes or better compatibilization of the films’ components (Cieśla & Abramowska, 2021). Li et al. (2018) developed and characterized gamma irradiated-corn-starch films. Corn starch was modified with irradiation doses of 10, 20, 30, and 40 kGy, with a dose rate of 16.7 ± 0.2 Gy min^−1^, and compared with native starch. The applied doses increased the tensile strength of the films, while the water vapor permeability decreased with the increasing doses. According to the authors, between all treatments, the 30-kGy irradiated corn starch film showed great potential for developing biodegradable starch film with improved properties.

Another study conducted by Cieśla & Abramowska, (2021) verified the effect of absorbed dose on starch:PVA (polyvinyl alcohol) films irradiated with gamma rays. In this case, the dose was applied directly to the film. They prepared films by casting the starch:PVA blends and irradiated them at 5, 10, 20, 30, 50, and 75 kGy doses. The irradiation with a dose of 5 kGy induced a decrease in surface hydrophilicity shown by an increase in contact angle to the water while no further impact was observed at higher doses. At doses higher than 20 kGy a decrease in films’ flexibility was obtained with no change in tensile strength. The authors also reported that a reduced gel hardness and increased solubility corroborating degradation prevails over crosslinking under gamma irradiation. In conclusion, the radiation modification carried out with gamma rays using doses up to 10 kGy may find application in manufacturing better films in the starch:PVA system. The elaborated films are suitable for packing foods.

### 5.2 Formation of starch based hydrogels under radiation

Hydrogels are formed by physically or chemically crosslinked macromolecules, forming a three-dimensional network capable of retaining water without disintegration (Ahmad et al., 2015). Their excellent network structure and substantial water absorption capacity make them suitable in many fields, including agriculture, personal hygiene, wound dressing, tissue engineering, drug delivery, and waste-water treatment (Bhuyan et al., 2016). Mainly hydrogels are prepared from the monomers crosslinked through physical interactions or chemical bindings induced via chemical reagents or ionizing radiations (Qamruzzaman et al., 2022). In this line, different types of high-energy radiations i.e. gamma radiation and electron beam are used to provoke grafing/crosslinking interactions into the gel lattice. Upon irradiation, the water and starch radical species produced throuh homolytic scission of C—H/O—H bonds induce covalent bonding among molecules faciltating the generation of a gel network. (Bhuyan et al., 2016, Nizam El-Din and Ibraheim, 2021, Qamruzzaman et al., 2022). The crosslinking promoted by irradiation is influenced by factors such as starch source, concentration, and type of radiation. For instance, Bhuyan et al., (2016) studied the synthesis of potato starch-acrylic-acid hydrogels under 5, 10, 15, 20, and 25 kGy of gamma irradiation and their application in dye adsorption. The authors obtained a higher swelling capacity when applied 15 kGy, and reported the radiation doses didn’t interfere with the decomposition temperature. Based on the adsorption results, the authors confirmed that hydrogels were efficient in adsorbing dye and can be applied on pharmaceutical, agricultural and environmental technologies.

In another attempt, Nizam El-Din & Ibraheim, (2021) elaborated nanocomposite hydrogels by gamma-radiation induced copolymerization of acrylic acid (AAc) onto plasticized starch (PLST)/montmorillonite clay (MMT)/chitosan (CS) blends and investigated the effect of irradiation dose and MMT content on the gel fraction and water absorption of the fabricated hydrogels. The adequate dose of gamma irradiation to achieve homogeneous hydrogel lattice and the highest water absorption was 15 kGy, regardless of composition. In addition, the authors tested the effect of the produced hydrogels on skin wound healing. The evaluated rat models revealed that wounds treated with the copolymer hydrogels healed faster, which may be a potential candidate for wound-healing dressing materials.

The effect of degree of substitution and irradiation on the properties of hydrogels prepared from carboxymethyl-sago starch and polyethylene glycol were investigated by Zahib et al. 2021. The obtained hydrogels showed that carboxymethyl-sago starch and polyethylene glycol hydrogels with substitution degree 0.4 give the ideal gel content when irradiated at 30 kGy. They concluded that radiation and polyethylene glycol addition improved most of the properties of carboxymethyl-sago starch irrespective of the substitution degree.

## 6 Impact of radiation on digestibility and nutritional attributes of starch

The digestibility of foods determines how effectively the gastrointestinal tract digests and absorbs the macronutrient (Welch, 2011). The molecular integration of food components, the existence of naturally occurring anti-nutritional agents, the food composition and thermal/non thermal processing of food, all have an impact on how quickly a component can be digested. Like most food processing methods, radiation techniques have been extensively examined and have the potential to cause various modifications that may alter the nutritional and chemical composition of foods. Upon such treatments, starches might become more/less digestible as a result of alterations in their structural integrity (fragmentation/breakdown). According to reports, gamma irradiation causes resistant starch to develop, decreasing the starch digestibility. The development of crosslinking, beta bonds, and carboxyl groups, which are resistant to enzyme assaults, is responsible for decreased digestibility (Rostamabadi et al., 2020, Bashir and Aggarwal, 2019, Sudheesh et al., 2019). For instance, (Lam et al., 2021) found an improve in indigestible fractions of rice starch treated with 10, 30, and 50 kGy Gamma irradiation. A similar decrease in digestibility has also been reported for rice (Khatun et al., 2021), corn (Lee et al., 2013), potato and bean (Chung & Liu, 2010) starches upon irradiation. However, (Sudheesh et al., 2019), conversely, reported an increase in digestibility of kithul (Caryota urens) starch under Gamma irradiation where increasing the irradiation dose from 0.5 to 10 kGy caused a decline in RS content from 51.23 to 41%. Authors stated that,the radiolysis depolymerization of starch molecules into fractions of lower molecular weight elevated their enzyme susceptibility. Moreover, the emerged superficial cracks and fissures increased the enzyme accessibility to deeper portions of starch granules, caused an enhance in their digestibility. In another attempt, (Govindaraju et al., 2022) obtained a drastic increase in amylase hydrolysis of potato, corn and rice starch from 1.66, 5.7, and 7.8 % to 10.66, 14.58, 17.03 %, respectively, when treated with Gamma irradiation. The starch digestibility was negatively correlated with the amylose content of starch indicating the loosened/cleaved amylose chains upon gamma irradiation makes them more prone to enzymatic actions. (Polesi et al., 2018) found a non-linear behavior in starch digestibility of rice starch when exposed to 1, 2, and 5 kGy gamma rays with an improve in RS and a decline in SDS at 1 kGy, no change in digestibility at 2 kGy and a final increase in SDS and a decrease in RS at the highest level of irradiation. Moreover, they outlined that the total dietary fiber (TDF) content were not affected by irradiation except for the specimens treated at 5 kGy gamma irradiation with an increase in TDF. (Jaiswal et al., 2021) applied 5 kGy gamma irradiation on pigmented and non-pigmented Sorghum starches and reported the radiolysis interactions and opening the granules’ structure caused a decrease in RS content of starches. Electron beam radiation has also shown to be a potent tool in enhancing the RS content of starch. For instance, in rice samples irradiated with low doses of EB (1–4 kGy) the RDS content decreased from 38.1 to 32.4 while, in contrast, the RS content elevated from 14.8 to 34.2. However, at higher doses, reverse trends were observed. This was in accordance with the changes in morphological attributes of granules and breakage of starch molecules at sever exposure to EB irradiation (Pan et al., 2020).

There are also numerous attempts investigating the impact of non-ionizing radiation on starch digestibility. In this line, (Xu et al., 2019) revealed that the morphological transformations of potato and maize starch granules upon high-power microwave treatment (6.63 w/g) changed their digestibility pattern where, the RS declined while SDS increased. Indeed, the appearance of hollows facilitated the entry of digestive enzymes, resulting in an elevated digestion. Y.-D. Li et al., (2018) invented a novel approach based on microwave-toughening to elevate the RS content of potato starch. They showed performing 100 s microwave (100 W) followed by toughening (55 °C for 16 h) and aging (4 °C for 18 h) could remarkably increase the RS from 11.54 to 27.09 %. This result ascribed to the partial degradation of starch molecules under MW treatment and their further rearrangement into well-ordered crystalline structures with less digestibility. The animal studies performed by these researchers revealed the compelling potential of modified starches in reducing the post-prandial blood glucose comparable to that of commercial type 4 resistant starch. The positive impact of MW on increasing the RS content of rice and sago starches has also been reported by (Cheng et al., 2022) and (Zailani et al., 2022), respectively. Nevertheless, on the contrary, MW might reduce the RS content of starch as it was reported by (Wang et al., 2022). This group studied the impact of MW treatment (480 W, 30–60 s) on in vitro digestibility of sweet potato starch and obtained a remarkable increase in RDS (from 19.98 to 29.41) and a decrease in RS (59.69 to 49.0). Such alterations were attributed to the elevated enzyme accessibility over morphological and structural changes of the starch granules. Overall, the changes in starch digestibility is influenced by a series of factors i.e. starch source, type of radiation, as well as radiation condition.

## 7 Challenges, limitations and concluding remarks

There has been an increasing interest towards the development of starches with modified morphologies and functionalities offering advantages over their conventional counterparts in various food and biomedical applications. Although ionizing/non-ionizing radiation treatments are proven to be rapid and effective techniques for improvement of nutritional and techno-functional properties of starches, there are still some issues that need to be addressed. The relatively high cost of radiation-assisted modification compared to the commonly used conventional techniques and the limited availability of the irradiation facilities contribute to the failure of their widespread industrial applications. In case of ionizing irradiation, the main limitation of irradiation treatment in food industrial applications is the toxicological hazards it imparts to food materials. In this regard various national and international agencies has been established to develop strict legislations for irradiated foods (e.g., the irradiation must be reflected on the label of the food, the irradiation facility must be approved by the food-relevant governmental agencies, and the list of foods that can be safely irradiated along with the maximum permitted dose of irradiation for each food must be available to the customers). Moreover, functional properties of starches treated by radiation are shown to be affected by the radiation type and treatment conditions applied. Besides, bioactivity of special interest such as antioxidant activity are indicated to be negatively affected by the irradiation dose (Criado et al., 2017). On the other hand, some functional properties including film forming and pasting or physical attributes such as color can be adversely influenced by the increasing doses of radiation. Hence, in future studies, the processing parameters need to be optimized for the desired functional attributes depending on the specific application area. In addition to radiation parameters, the nature, botanical origin, structure and composition of the material are indicated to be effective parameters governing the changes in functionality. Thus, precise control of these parameters was suggested to obtain desired characteristics and novel functionality in the modified starches. Furthermore, combination of radiation with other non-thermal processing techniques such as ultrasound or high-pressure processing can provide more in-depth information related to the improvement of nutritional and functional properties of starch for specific applications. From the nutritional point of view, gamma irradiation of starches was not considered as the most recommended radiation technique due to the production of acids, sugars, and free radicals upon processing. Hence, standardization of gamma irradiation dose for targeted functionality and quality was indicated as an area that needs to be addressed in future studies. Another challenge associated with the use of radiation-modified starches is the possible degradation of backbone polymers under the effect of ionizing radiation which may reduce the mechanical and chemical stability, hence limiting their practical application. Application of cross-linking is suggested as an effective strategy for overcoming the polymer degradation. Although effects of ionizing and non-ionizing radiation treatments on functional and nutritional properties of various starches are investigated extensively in recent studies, there is yet limited research on the application performance of modified starches. Further research is needed to explore the applicability of radiation-modified components in novel and existing formulations and their effects on product quality and performance.

## Declaration of Competing Interest

Dr. M.R. Nemțanu acknowledges that her contribution to this work was supported by a grant of the Romanian Ministry of Research, Innovation and Digitalization, CNCS - UEFISCDI, project number PN-III-P4-PCE-2021-1778, within PNCDI III.

## Data Availability

Data will be made available on request.
